# Exploratory investigation of pain threshold in the facial and cervical regions following dental implant surgery

**DOI:** 10.1007/s10006-026-01584-y

**Published:** 2026-06-24

**Authors:** Kiyomi Uehashi, Shoichi Ishigaki, Ryota Takaoka, Daisuke Moriguchi, Hirofumi Yatani

**Affiliations:** Department of Regenerative Prosthodontics, Graduate School of Dentistry, The University of Osaka, 1-8 Yamadaoka, Suita, 565-0871 Osaka Japan

**Keywords:** Dental implant surgery, Pain threshold, Thermal stimulation, Subclinical sensory changes, Trigeminal nerve

## Abstract

**Purpose:**

The pathogenic mechanisms and variations in individual pain susceptibility underlying neuropathic pain associated with dental implant surgery have not been fully elucidated, and objective evaluation methods for such pain remain unestablished. The objective of this study was to identify exploratory trends in subclinical sensory changes by evaluating changes in pain thresholds following dental implant surgery.

**Methods:**

The subjects included 76 outpatients who visited the Department of Fixed Prosthodontics at Osaka University Dental Hospital for dental implant surgery. Pain thresholds were recorded before and after surgery within the regions innervated by the second and third divisions of the trigeminal nerve and the upper cervical plexus. Measurements were performed using a computer-controlled thermal sensory analyzer (PATHWAY, Medoc Co. Ltd).

**Results:**

The decline of the pain threshold event detected by the thermal stimulation on the operating side occurred in 28.57–48.57%, 10.48–25.90%, 1.40–10.36% and 0.09–1.38% of subjects at 1, 2, 4, and 12 weeks post-surgery, respectively.

**Conclusions:**

This prospective cohort study evaluated the impact of dental implant surgery on pain thresholds. The findings suggest that a significant proportion of patients may experience transient post-surgical sensory changes, characterized by a decline in their pain threshold following surgery, even in the absence of subjective pain complaints.

## Introduction

Pain is defined as “An unpleasant sensory and emotional experience associated with actual or potential tissue damage, or described in terms of such damage” [1]. Neuropathic pain, a pathological condition arising from peripheral nerve injury, dysfunction, and/or maladaptive plastic changes in the central nervous system, often follows a chronic course. It is commonly triggered by conditions such as malignancy, diabetes, traumatic injury, and post-surgical complications [2].

Chronic post-surgical pain (CPSP) has been reported in 20% to 50% of systemic surgery cases [3] and 5% to 13% of dental procedures [4]. Although numerous cases of neuropathic pain have been documented [5, 6], its specific prevalence within the context of chronic post-operative pain remains largely unreported.

In the orofacial region, chronic pain can be triggered by routine treatments, including tooth extraction [7, 8], pulp extirpations [9], endodontics therapy [10–12] and dental implant placement [13, 14]. Neuropathic pain predominantly stems from nerve injuries sustained during implant surgery [13–17]; many of these cases are highly intractable, causing patients to experience severe, debilitating pain [18]. Despite its clinical significance, the underlying pathogenic mechanisms have not been fully elucidated, and standardized objective evaluation methods remain unestablished [19].

To evaluate and diagnose neuropathic pain, sensory and pain intensities are typically assessed using electrical, mechanical (pressure/vibration), or thermal stimuli [19, 20]. Recent diagnostic protocols for neuropathic pain allow for a comprehensive integration of these examination methods [21–23]. Among these Quantitative Sensory Testing (QST) modalities, thermal stimulation devices have been refined for their repeatability and ease of use, making them a primary recommendation for clinical examinations globally [24].

The present prospective exploratory study utilizes QST with thermal stimulation in patients undergoing dental implant surgery. We investigated the incidence of decreased pain thresholds and the specific localization of post-operative pain to gather objective data, aimed at characterizing the experimental subclinical sensory changes and transient sensitization following surgery.

## Material and method

### Subjects

Among 105 outpatients who visited Osaka University Dental Hospital for dental implant surgery, 76 subjects were enrolled based on the following inclusion criteria: (1) planning to undergo implant surgery, and (2) aged 20 years or older. The exclusion criteria were: (1) cutaneous diseases in the orofacial region; (2) pre-existing pain or paralysis in the orofacial region; and (3) females taking oral contraceptives [25]. The final cohort consisted of 24 males (range: 20–74 years; mean ± SD: 55.9 ± 16.3 years) and 52 females (range: 21–77 years; mean ± SD: 55.7 ± 14.0 years).

**Fig. 1:**
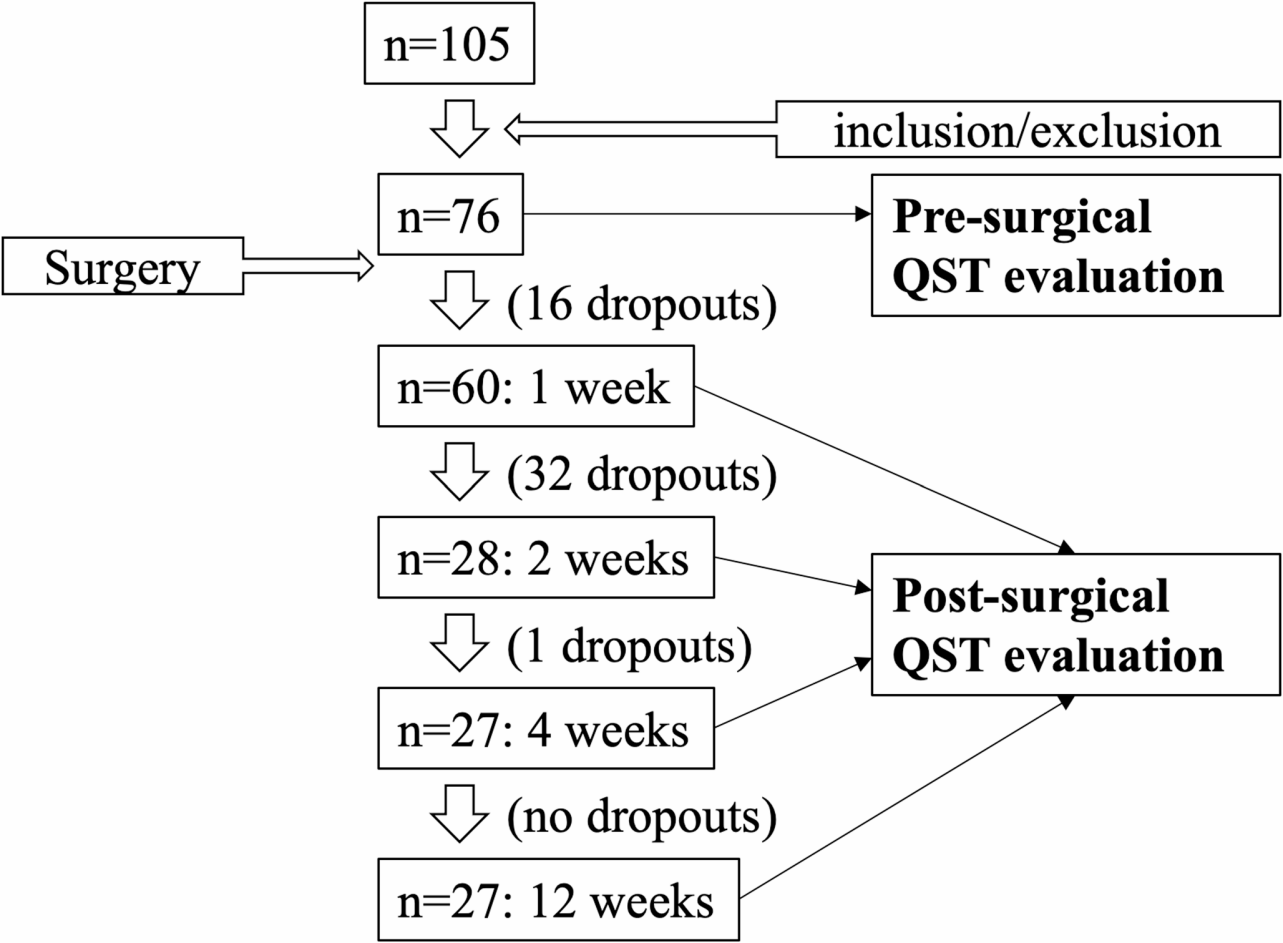
Study samples

During the follow-up period, the following dropouts occurred: at one week post-surgery, 16 subjects withdrew (14 declined further participation, and two could not be measured due to severe post-operative pain). At two weeks, an additional 32 subjects refused further measurements. By four weeks, one more subject withdrew, leaving 27 subjects for the final analysis (Fig. 1).

Implant placement locations included 35 mandibular (21 unilateral, 14 bilateral), 22 maxillary (18 unilateral, 4 bilateral), and three maxillomandibular cases (one unilateral, two bilateral). To avoid double-counting bias, the three maxillomandibular cases were excluded from specific site analyses. A total of 162 implants (range: 1–8 per patient; mean: 2.7) were placed using the Brånemark System^®^ or Replace^®^ Select (Nobel Biocare A/S).

No significant differences in age, sex, or the location and number of implants were found between the dropout group and the remaining participants (Table 1), confirming that the dropouts did not introduce selection bias. This study was approved by the Institutional Review Board of the Osaka University Graduate School of Dentistry (#H18-16), and written informed consent was obtained from all subjects.

**Table 1:** Patients characteristics before and after the dropouts

	Before surgery (*n* = 76)	1week(*n* = 57)	12week(*n* = 27)	*p*-value
Age	55.9 ± 13.8	55.5 ± 13.1	55.3 ± 13.9	0.85^†^
Sex (male/female)	24 / 52	18 / 39	7 / 20	0.85*
Location of implant placement	34 / 42	22 / 35	10 / 17	0.69*
The number of implant placements	2.7 ± 1.5	2.6 ± 1.5	2.9 ± 1.7	0.95^†^

^†^: ANOVA (Bonferroni correction), *: χ^2^ test

### Pain threshold measurements

**Fig. 2:**
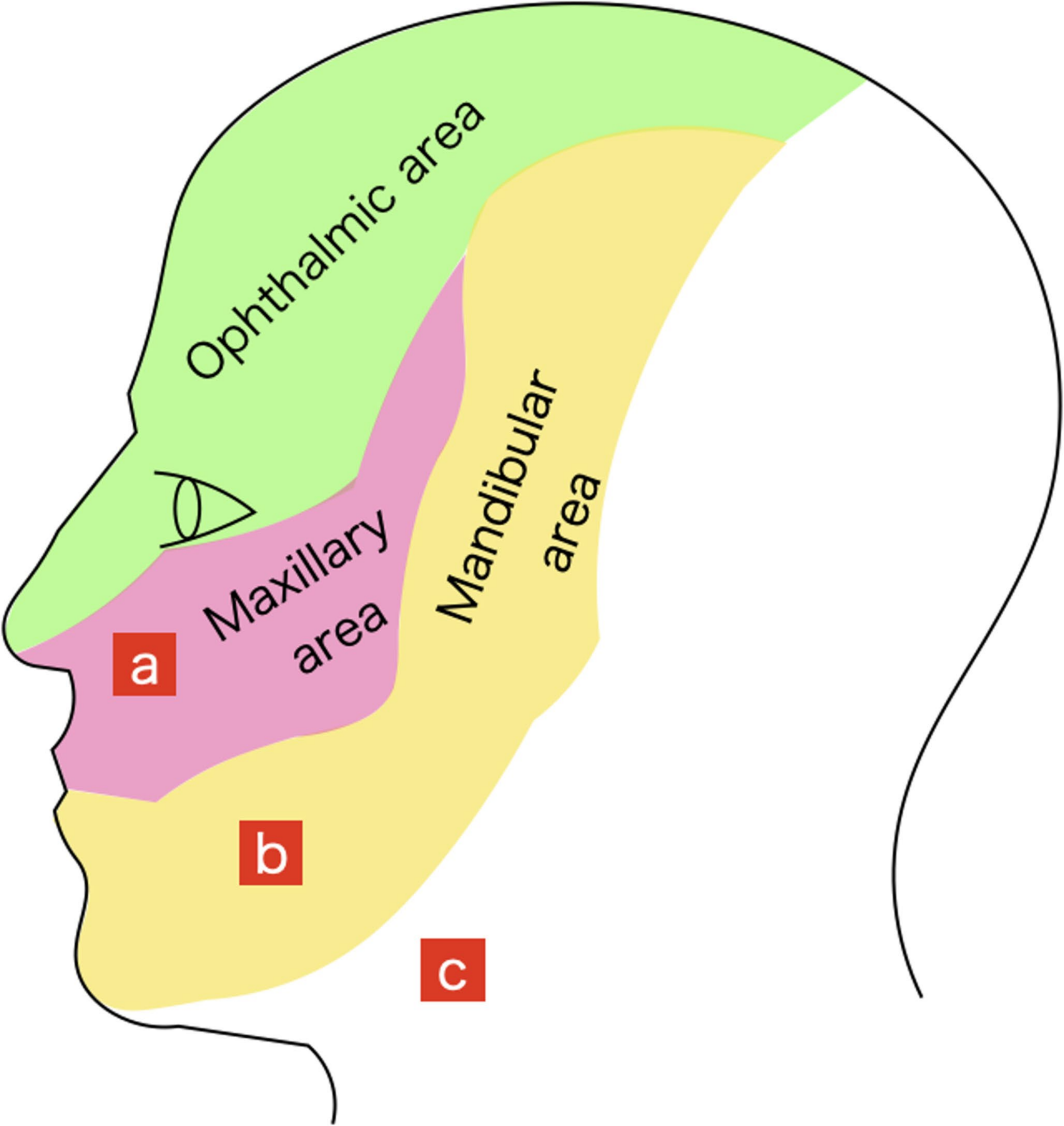
Recorded site. **a**: Recorded site on trigeminal nerve second branch (V2). **b**: Recorded site on trigeminal nerve third branch (V3). **c**: Recorded site on upper cervical nerve distribution area (C)

Pain thresholds were evaluated using a computer-controlled thermal sensory analyzer (PATHWAY, Medoc Co. Ltd.) in regions innervated by the trigeminal nerve (V2 and V3) and the upper cervical plexus (C). Measurements were performed bilaterally at the following standardized sites, with the brachial cutaneous surface serving as a control:

- V2: A point 2 cm from the ala nasi on the line toward the tragus.
- V3: A point 2 cm from the angle of the mouth on the line toward the gonion.
- C: A point 2 cm below the midpoint between the menton and the gonion (Fig. 2).

The PATHWAY system utilizes a 16 mm × 16 mm thermode (baseline 32 °C; range − 15 °C to 55 °C) to selectively stimulate Aδ and C fibers by modulating the temperature change rate [26, 27]. Subjects pressed a response button immediately upon perceiving pain, triggering the thermode to return to baseline. The measurement sequence included:

- Fast Heat (FH): Temperature increased at 2.5 °C/s to selectively stimulate Aδ fibers.
- Slow Heat (SH): Temperature increased at 1.0 °C/s to selectively stimulate C fibers.

Assessments were conducted before surgery (baseline), and at one week, two weeks, one month, and three months post-surgery. All recordings were performed by the same examiner in a temperature-controlled room (25 °C). A “decline of the pain threshold event” was defined as a decrease of more than 1 °C from the baseline.

### Subjective pain intensity

Subjective pain intensity was recorded immediately after surgery and at each follow-up interval using a 100-mm Visual Analog Scale (VAS). The scale was anchored by “no pain at all” (0 mm) and “worst pain imaginable” (100 mm).

### Statistical analysis

Patient characteristics between the dropout and non-dropout groups were compared using Chi-square tests and ANOVA with Bonferroni correction. Changes in pain thresholds over time were analyzed using Dunnett’s test.

Subjects were categorized into the “threshold decline” group (> 1 °C decrease) or the “no-decline” group. Event rates for FH and SH were calculated using the Kaplan-Meier method for both surgical and non-surgical sides. The curve illustrated the proportion of patients exhibiting a decline in pain threshold relative to the total cohort, with the disappearance of the threshold decline serving as the endpoint. Although Kaplan–Meier analysis is traditionally applied to irreversible events, we utilized this approach as an exploratory mean to visually and chronologically track the initial onset and maintenance tendency of threshold decline events post-surgery.

## Results

### Pain thresholds in the V2 innervation for maxillary placements (Fig. 3)

**Fig. 3:**
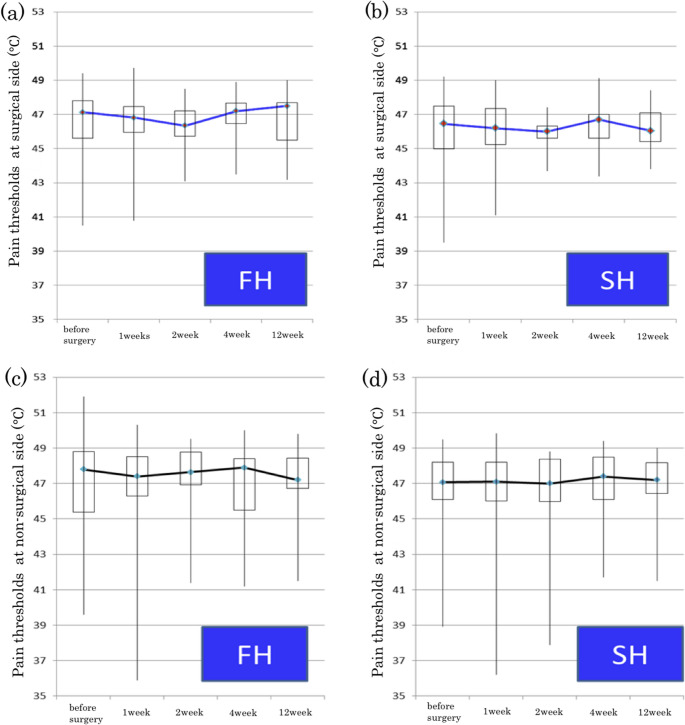
Pain thresholds in the V2 innervation for maxillary placements. **a**: Operative side FH thresholds, **b**: Operative side SH thresholds, **c**: Non-operative side FH thresholds

For patients with bilateral placements, mean scores for the right and left sides were calculated for analysis. Although not statistically significant, operative side FH thresholds showed a decline two weeks post-surgery, followed by recovery at 4 and 12 weeks. On the non-operative sides of the V2 innervations, no decline in pain thresholds was detected. Thresholds for both FH and SH did not differ significantly from baseline at any post-operative interval.

### Pain thresholds in the V3 innervation for mandibular placements (Fig. 4)

**Fig. 4:**
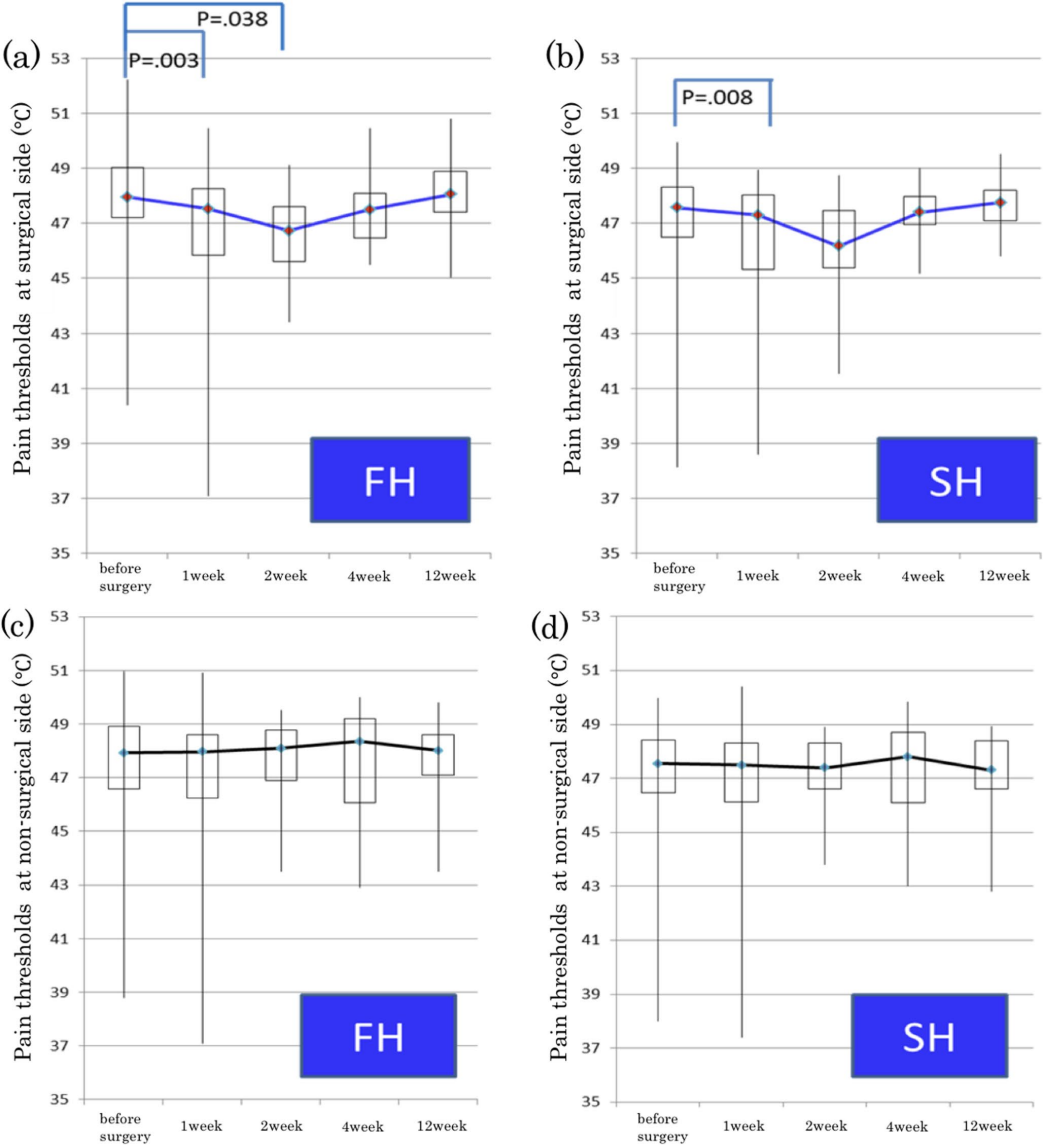
Pain thresholds in the V3 innervation for mandibular placements. **a**: Operative side FH thresholds, **b**: Operative side SH thresholds, **c**: Non-operative side FH thresholds

FH thresholds on the operative side significantly declined one week (*P* = 0.003) and two weeks (*P* = 0.008) post-surgery. Similarly, SH thresholds significantly declined at the one-week interval (*P* = 0.008). On the non-surgical sides of the V3 innervations, no decline in pain thresholds was detected. Thresholds for both FH and SH did not differ significantly from baseline at any post-operative interval.

### Pain thresholds in the C innervation (Fig. 5)

**Fig. 5:**
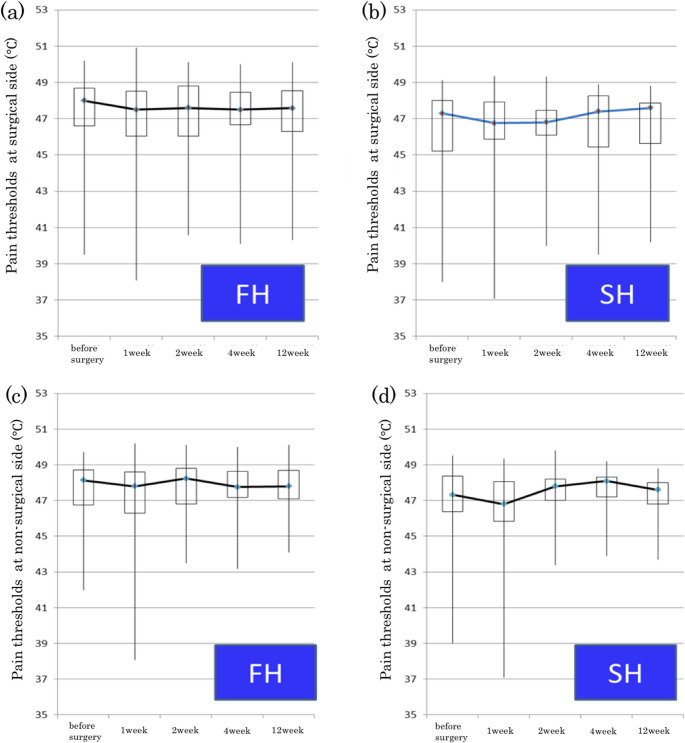
Pain thresholds in the C innervation. **a**: Operative side FH thresholds, **b**: Operative side SH thresholds, **c**: Non-operative side FH thresholds

Regarding the thresholds in the C innervation, mean scores from both sides were analyzed for both maxillary and mandibular placements. No decline of the pain thresholds was seen. No significant decline in pain thresholds was observed; thresholds for both FH and SH remained stable throughout the pre- and post-operative periods. On the non-surgical sides of the C innervations, no decline in pain thresholds was detected. Thresholds for both FH and SH did not differ significantly from baseline at any post-operative interval.

### Temporal change of the decline events of the pain threshold by maxillary placements (Fig. 6)

**Fig. 6:**
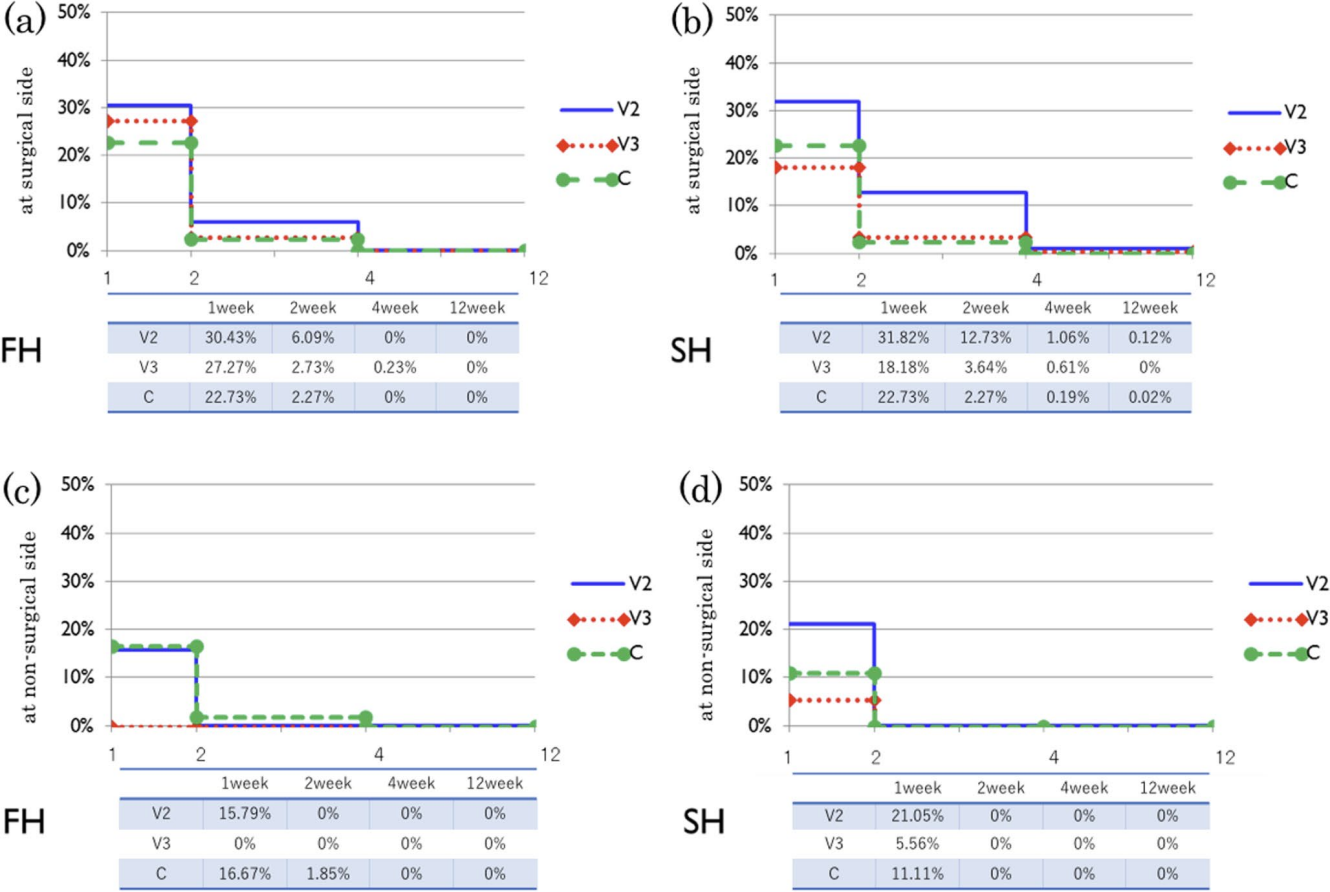
Temporal change of the decline events of the pain threshold by maxillary placements. **a**: Operative side FH thresholds, **b**: Operative side SH thresholds, **c**: Non-operative side FH thresholds

Maxillary placements one week post-surgery, operative-side FH threshold decline events occurred in 30.43% of the V2, 27.27% of the V3, and 22.73% of the C regions. While these events were prevalent across all regions at one week, they were absent by 12 weeks (Fig. 6a). SH threshold decline events at one week were observed in 31.82% of the V2, 18.18% of the V3, and 22.73% of the C regions. By 12 weeks, these events persisted only minimally (0.12% in V2 and 0.02% in C regions). The frequency of threshold decline was slightly higher at surgical sites compared to adjacent sites (Fig. 6b).

On the non-operative side, FH threshold decline was observed in 15.79% of the V2 and 16.67% of the C regions at one week, with no events recorded after four weeks (Fig. 6c). For SH, the incidence at one week was 21.05% in V2, 5.56% in V3, and 11.11% in C regions, with complete resolution thereafter (Fig. 6d). Overall, the frequency of threshold decline was lower on the non-operative side, with total resolution occurring within four weeks.

### Temporal change of the decline events of the pain threshold by mandibular placements (Fig. 7)

**Fig. 7:**
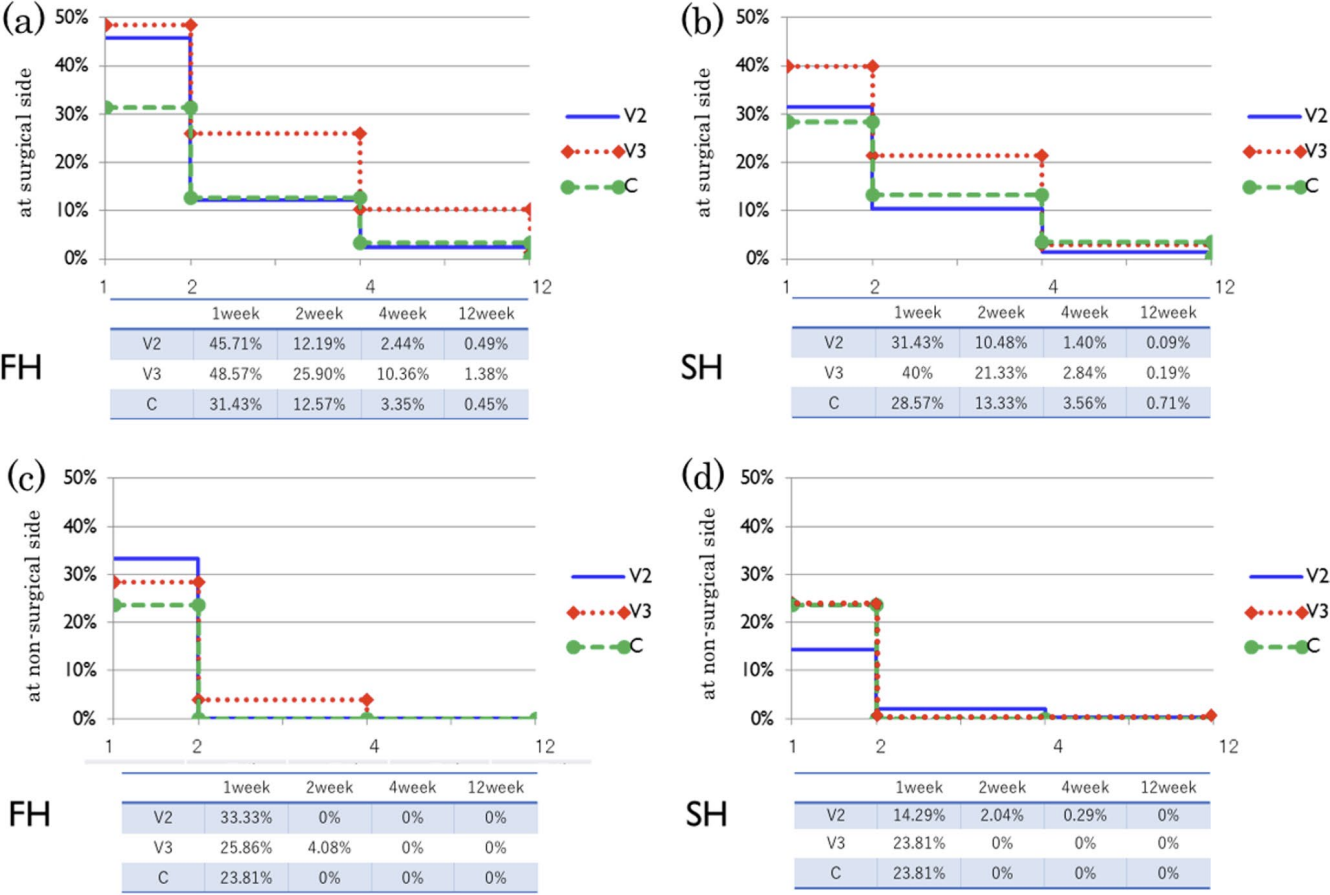
Temporal change of the decline events of the pain threshold by mandibular placements. **a**: Operative side FH thresholds, **b**: Operative side SH thresholds, **c**: Non-operative side FH thresholds

FH threshold decline events at one week were observed in 45.71% of the V2, 48.57% of the V3, and 31.43% of the C regions. These events occurred in all innervations at this interval. The incidence gradually decreased by two weeks (V2: 12.19%, V3: 25.90%, C: 12.57%) and four weeks (V2: 2.44%, V3: 10.36%, C: 3.35%). Occasional events persisted at 12 weeks (V2: 0.49%, V3: 1.38%, C: 0.45%; Fig. 7a). For SH, the incidence at one week was 31.43% in V2, 40.00% in V3, and 28.57% in C regions. While the frequency declined rapidly, sporadic events were still recorded at 12 weeks (V2: 0.09%, V3: 0.19%, C: 0.71%; Fig. 7b). Notably, threshold decline was more frequent in mandibular than in maxillary placements. On the non-operative side, FH events at one week occurred in 33.33% of the V2, 25.86% of the V3, and 23.81% of the C regions, with no events observed beyond that point. SH events at one week were observed in 14.29% of the V2 and 23.81% of both the V3 and C regions, followed by complete resolution by 12 weeks. The incidence of these events was consistently lower on the non-operative side. Temporal change of the decline of the pain threshold events at the brachial cutaneous surface for control. Threshold decline events occurred in 8.33% of FH and 2.78% of SH measurements at one week, with total resolution by 12 weeks.

### Temporal change of the decline events of the pain threshold at the brachial cutaneous surface for control (Fig. 8)

**Fig. 8:**
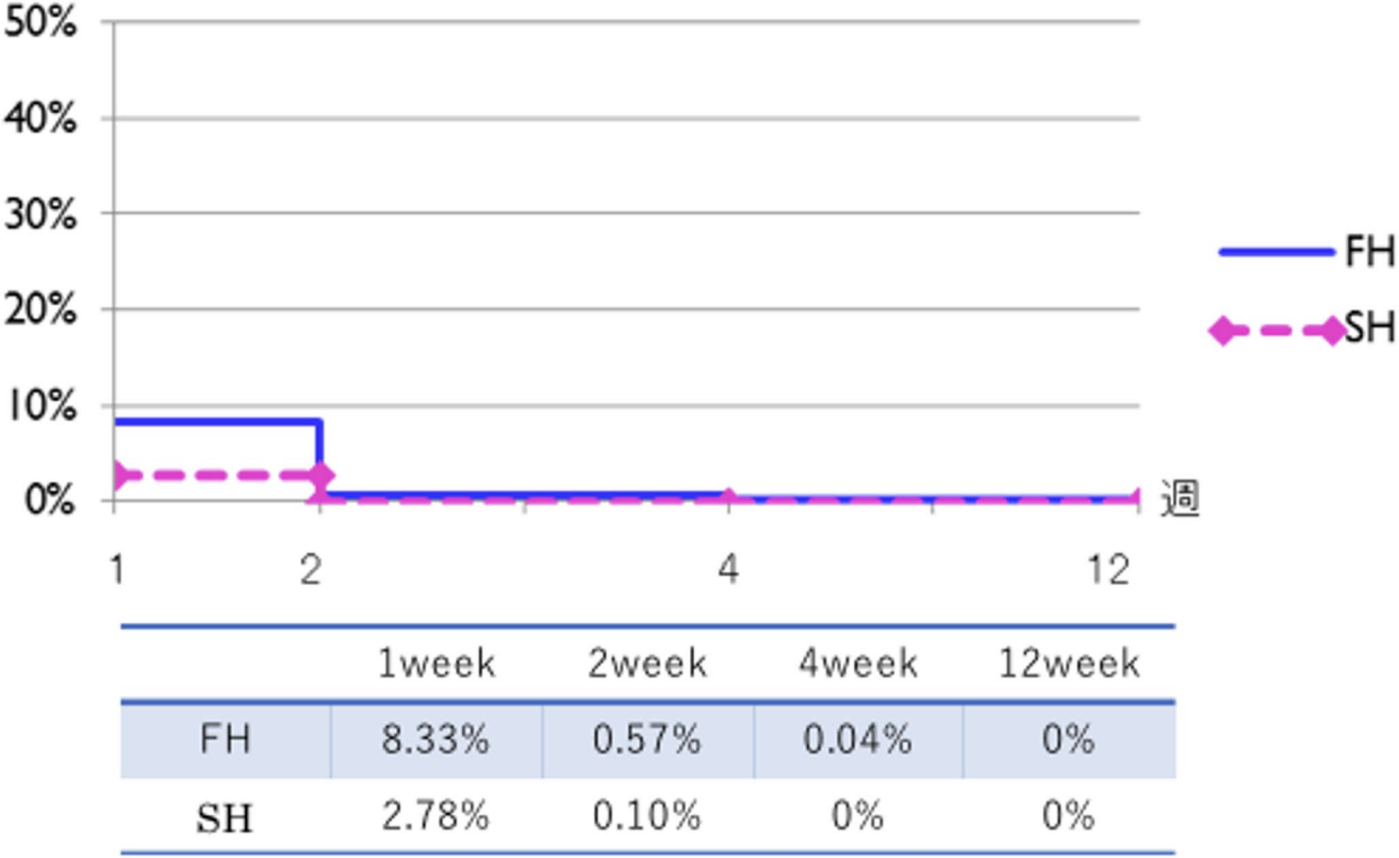
Temporal change of the decline events of the pain threshold at the brachial cutaneous surface for control

Brachial cutaneous control site at the brachial control site, threshold decline events occurred in 8.33% of FH and 2.78% of SH measurements at one week, with no events recorded by 12 weeks.

### Assessment of the subjective pain intensity (Fig. 9)

**Fig. 9:**
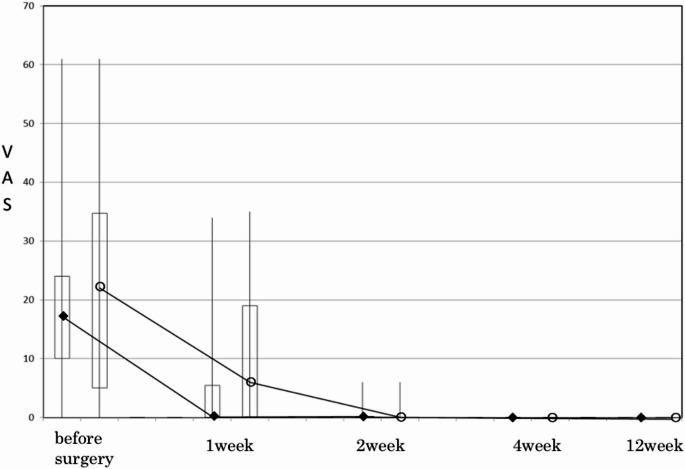
Assessment of the subjective pain intensity

There was no significant difference in subjective pain intensity (VAS scores) between the “threshold decline” and “no-decline” groups at any post-operative interval (*P* = 0.538;). Furthermore, no subjects reported pain beyond four weeks post-surgery. All participants discontinued analgesic medication within one day of the procedure.

## Discussion

In clinical dental practice, although routine procedures such as endodontic therapy, extractions, and implant placements may cause trauma to the terminal branches of the trigeminal nerve, reported cases of orofacial neuropathic pain remain relatively scarce [28]. However, a lack of comprehensive understanding of neuropathic pain among clinicians may lead to frequent underdiagnosis [29–31]. In the present study, implant placement referred specifically to the first stage of a two-stage surgical protocol, encompassing infiltration anesthesia, incision, mucoperiosteal flap elevation, osteotomy, fixture placement, and suturing.

An objective gold standard for pain assessment has yet to be established [32]. Quantitative Sensory Testing (QST) assesses neural function across the peripheral and central nervous systems through thermal, vibration, pressure, and electrical stimuli [20, 21]. We utilized thermal threshold testing due to its high reproducibility and simplicity, although we acknowledge that results can be influenced by skin thickness and core body temperature. Cold nociception was excluded from our protocol due to high inter- and intra-individual variability [33, 34]. Unlike electrical stimuli, thermal stimulation via the PATHWAY system targets specific sensory receptors, primarily activating Aδ and C fibers [24]. Warm sensations are typically mediated by unmyelinated (C) [35] and small myelinated (Aδ) fibers, with faster rates of temperature change predominantly activating the latter [26, 27]. Accordingly, we set the thermal change rates to 2.5 °C/s (FH) for Aδ fiber stimulation and 1.0 °C/s (SH) for C fiber stimulation.

A “decline in pain threshold” was defined as a decrease of more than 1.0 °C. While the reported differential threshold in the facial region is 0.2–0.4 °C [36], we selected a 1.0 °C threshold to account for potential response time lags between pain perception and button activation.

For maxillary placements, no significant changes in V2 thresholds were observed with either FH or SH. Conversely, in mandibular cases, FH thresholds declined significantly at one and two weeks post-surgery, and SH thresholds declined at one week. This discrepancy suggests that the mandibular nerve may be more susceptible to injury or sensitization during dental procedures than the maxillary nerve [37]. Specifically, a decline in thresholds occurred in approximately 30% of maxillary and 50% of mandibular cases one week post-surgery, suggesting the occurrence of transient peripheral sensitization. This sensitization is likely driven by increased nociceptor excitability resulting from local inflammation [38, 39].

Importantly, pain thresholds declined even in subjects who did not report subjective pain. While post-operative pain intensity is often cited as a primary risk factor for chronic pain [3, 4, 40–42], our results showed no significant difference in subjective VAS scores between the “threshold decline” and “no-decline” groups. Subjective pain was generally mild and largely resolved within one week. The observation that threshold declines persisted long-term and extended beyond the immediate surgical site suggests that central sensitization, in addition to peripheral mechanisms, may be involved [43, 44]. However, since our study design does not support definitive causal inference, these findings should not be overinterpreted as conclusive evidence of implant-induced neuropathic pain mechanisms. Future research must determine whether these subclinical sensitizations are definitive precursors to chronic neuropathic pain.

The study initially enrolled 76 subjects; however, the cohort size decreased to 60 at one week, 28 at two weeks, and 27 at four weeks post-surgery, remaining stable thereafter. Despite this notable attrition, a comparison of baseline characteristics between the remaining participants and those who dropped out revealed no significant differences in age, sex, or the location and number of implants (Table 1). This suggest that the dropouts did not introduce significant selection bias. However, this high dropout rate represents a major limitation of this study, as it significantly limits the robustness of our results. Specifically, the substantial loss of participants over time may have reduced the statistical power to detect subtle, long-term chronological changes and potentially compromised the internal validity due to attrition bias. Consequently, these factors may restrict the generalizability of our findings to a broader clinical population, meaning that the results, particularly at the 12-week follow-up, must be interpreted with caution.

The definition of “threshold decline” (> 1 °C) used as a primary parameter in this study requires careful interpretation. This parameter is not an established surrogate marker for neuropathic pain, and its clinical significance remains unclarified. Therefore, we acknowledge the exploratory nature of this threshold and avoid presenting it as a definitive, clinically meaningful endpoint. Furthermore, the application of Kaplan–Meier analysis to these threshold decline events has methodological limitations. Because Kaplan–Meier estimation assumes the events to be irreversible, it does not fully account for the reversible and fluctuating nature of the somatosensory changes observed in our participants. Consequently, while it effectively illustrates the cumulative timeline of initial threshold shifts, the statistical curves must be interpreted with caution regarding the long-term resolution of sensitization. In addition, we observed a lack of association between the QST findings and subjective pain scores (VAS), and no persistent pain was reported among the participants. This discrepancy suggests that the observed changes in pain thresholds may reflect subclinical or transient neurophysiological alterations rather than clinically manifest neuropathic pain. While QST is highly sensitive in detecting subtle, micro-level somatosensory variations following dental implant surgery, its clinical relevance as a direct surrogate marker for subjective or persistent pain remains limited. Further well-designed clinical studies are required to validate the clinical relevance and diagnostic validity of this specific threshold decline in evaluating somatosensory changes following dental implant surgery.

If a declining pain threshold reflects a temporary subclinical sensitization, the degree of surgical trauma may serve as a critical risk factor. While quantifying surgical invasion is complex, it is likely that the risk is multifactorial, involving biological and environmental components. In particular, substantial heterogeneity existed in our cohort regarding the number of implants, their specific anatomical locations, and individual surgical characteristics, which may have introduced confounding variations in the degree of tissue trauma. Due to the small sample size resulting from high attrition at later time points, we were unable to perform robust adjusted multivariate analyses to control for these surgical variables. This heterogeneity represents a limitation in isolating the exact determinants of threshold shifts. As the exploratory study to quantitatively track temporal changes in pain thresholds following implant surgery, these findings provide essential experimental data on post-surgical somatosensory processing and transient subclinical sensitization.

## Conclusion

This study represents an exploratory prospective analysis to evaluate pain threshold changes following dental implant surgery using QST. Our findings indicate that transient sensory changes, manifesting as a decline in pain thresholds occurs at both surgical and adjacent sites, which may be mediated by peripheral and central sensitization, even in patients who experience clinically unremarkable post-operative recovery. Despite the absence of subjective pain complaints, subclinical sensitization was detectable. Given the increasing prevalence of dental implant procedures, understanding these post-operative sensory phenomena is vital. Further research is warranted to investigate whether these early sensory changes are related to the long-term development of chronic sensory disturbances.

## Data Availability

The datasets generated and/or analyzed during the current study are not publicly available because we do not possess any freely accessible cloud storage but are available from the corresponding author on reasonable request.
